# Antibiomania: A Rare Case of Metronidazole-Induced Mania

**DOI:** 10.7759/cureus.12414

**Published:** 2021-01-01

**Authors:** Piyush Puri, Pankul Parnami, Akshit Chitkara, Pal Satyajit Singh Athwal, Sunil Khetrapal

**Affiliations:** 1 Internal Medicine, Al-Falah School of Medical Sciences & Research Center, Faridabad, IND; 2 Gastroenterology, BLK Super Speciality Hospital, New Delhi, IND; 3 Internal Medicine, Shri Moolchand Super Speciality Hospital, Karnal, IND; 4 Internal Medicine, Saraswathi Institute of Medical Sciences, Hapur, IND; 5 Internal Medicine, Sunil Khetrapal's Hospital, New Delhi, IND

**Keywords:** metronidazole, mania, bipolar, bipolar disorders, psychosis, neurotoxicity, antibiomania, drug, psychiatry, hyperammonemia

## Abstract

Metronidazole is a very commonly used drug for the treatment of ailments caused by bacteria and parasites. It can treat a vast array of conditions like rosacea, sexually transmitted diseases (STDs), liver abscess, bedsores, etc. Metronidazole comes with generic side-effects like nausea, vomiting, dizziness, metallic taste, and also rare side-effects like paresthesia, syncope, cerebellar symptoms, psychosis but mania is a rare side-effect. Here, we present a case of metronidazole induced mania in a 50-year-old male with no past medical history who initially presented with a complaint of mild fever, loss of appetite, and fatigue from the past 10-12 days. On further examination and investigations, diagnosis of the amebic liver abscess was made on the basis of USG, serum serology for amebiasis IgG, and a CT scan. Consequently, the patient was started on the drug of choice for amebic liver abscess; IV metronidazole 1.5g/day divided over the day into three doses. Other drugs that were administered were pantoprazole, paracetamol, and ondansetron. On the ninth day of admission, the patient's wife as well as the physician-daughter of the patient reported a change in the behavior of the patient which became a major concern for the family. The patient was restless, energetic, unable to sleep, had racing thoughts, elated mood, petulant, and kept singing loudly in the private patient room. There was no history of any psychiatric illness in the family. Mr. K´s manic symptoms were managed using haloperidol and lorazepam. Upon discontinuing metronidazole, there was a gradual improvement in the manic symptoms, and symptoms improved, haloperidol and lorazepam were able to be tapered down and eventually stopped. Mr. K did not require any use of any selective serotonin reuptake inhibitor (SSRIs), monoamine oxidase inhibitors (MAOIs), serotonin-norepinephrine reuptake inhibitors (SNRIs), tricyclic antidepressants (TCAs), or any other atypical psychotropic drug. Manic-psychosis side-effect is a rare entity caused by antibiotics and the symptoms of which would disappear in a few days after stopping the antibiotic. It is also notable that this patient recovered without the use of any psychotropic drugs. Physicians should be aware of the possible neuropsychiatric side-effects of antibiotics which can lead to unnecessary workup. This side-effect did not require the use of any psychotropic drugs in this patient.

## Introduction

Metronidazole (nitroimidazole derivative of a Streptomyces) antibiotic was first used in 1959 for treating trichomoniasis, a common form of sexually transmitted disease (STD) [1]. Metronidazole is known to cause CNS side effects like paresthesia, cerebellar symptoms, syncope, confusion, psychosis, encephalopathy, etc. [2-4]. Metronidazole is one of the rare drugs which was initially meant to be used against parasites but it reaped popularity for its antibacterial action principally on anaerobic bacteria like Bacteroides, Fusobacterium spp, Peptostreptococci, and Clostridia spp [5]. It was in the year 1962 when parasitic trichomonal vaginitis infested patient treated with metronidazole was also cured of bacterial gingivitis [6]. 

Antidepressant-induced mania is a commonly known phenomenon in patients with bipolar disorder and patients can have mood changes when on acute anti-depressant therapy [7]. Drug-induced mania is also reported otherwise in patients taking non-psychotropic drugs and with no previous psychiatric history. Drugs like steroids, sympathomimetic amines (amphetamine, phenylephrine), iproniazid, levodopa, thyroxine, isoniazid, alprazolam, captopril, baclofen were amongst the drugs causing manic episodes [8,9]. Apart from these, antimicrobials are also known to cause manic episodes. Antibiotics like clarithromycin, erythromycin, cotrimoxazole, ciprofloxacin, ofloxacin, have been reported to cause manic syndromes in patients [10]. These antibiotics are very commonly used in today´s era but manic-psychotic side-effects of these drugs remain unestablished and enlightening the physicians about it is a major concern else it could lead to unnecessary treatment and workup. 

Manic episodes related to metronidazole are further super-rare and less than 10 instances have been reported [11, 12]. Antibiomania is a rare phenomenon and physicians should be aware of new-onset manic-psychic symptoms that might be caused by the routine drugs.

## Case presentation

Mr. K, a 50-year-old patient with no past medical history came with a complaint of mild fever, loss of appetite, and fatigue from the past 10-12 days, and was admitted to the hospital. The patient's blood pressure was 120-130/70-80 mmHg and his pulse rate was 70-80/min. Mr. K is a non-alcoholic, a non-smoker, and a vegan. Further lab tests were conducted which showed normal blood electrolytes, lipid profile, blood sugar, renal function tests were with normal ranges, but there was an increase in the AST, ALT, and neutrophil count.

A standard broad-spectrum antibiotic, amoxicillin with clavulanic acid is administered IV but with no good response. Subsequently, the USG study was done which showed an abscess which was followed by amebic serum serology for IgG which turned out to be positive.

**Figure 1:**
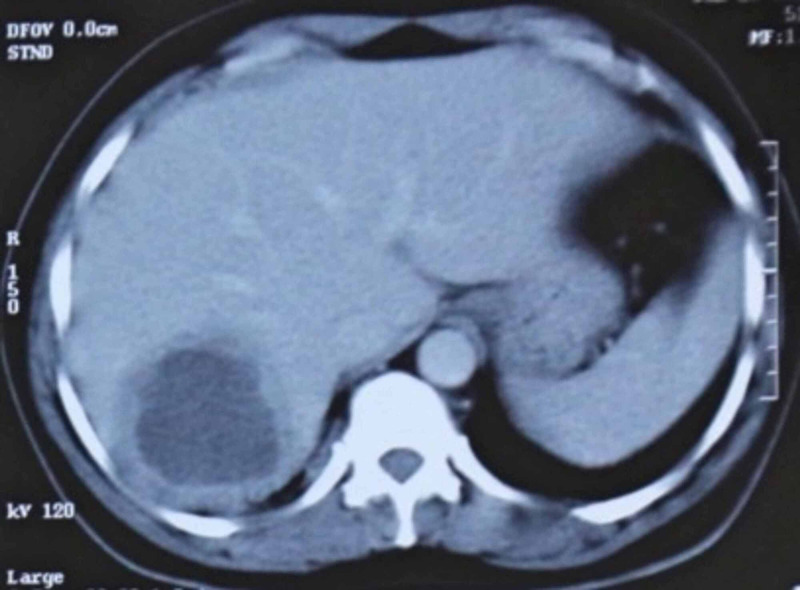
CT abdomen showing the amebic abscess in the right lobe of the liver.

Amebic liver abscess was additionally confirmed with a CT scan of the thorax and the abdomen to confirm the findings which showed the evidence of amebic liver abscess (Figure 1). Now, the treatment for a non-complicated hepatic liver abscess in developing countries is to manage it conservatively with metronidazole. The patient was administered IV Metronidazole, 500mg T.D.S i.e., 1.5g/day. Mr. K´s fever, fatigue, and appetite were responding deftly but on the 9th day of admission, the patient´s wife and the physician-daughter reported major changes in the behavior of the patient. The patient was talking excessively, singing loudly, unable to sleep, poor judgment with money, sexually inappropriate, and euphoric. Memory, orientation, and other higher mental functions were normal. There´s no history of photophobia, neck rigidity, seizure, or concealed or overt bleeding. MRI of the brain was done which was not significant and showed no focal or any space-occupying lesions. EEG came out to be normal too.

The patient was managed with haloperidol and lorazepam. Lab investigations were repeated which showed hyperammonemia and mild derangement in the LFTs. Initially, hyperammonemia was thought to be the cause of the change in the behavior. To treat hyperammonemia, all the drugs were stopped once and lactulose was administered. The very next day ammonia levels were reduced and were within the normal reference range. Also, the psychiatric symptoms of the patient improved gradually.

Now, the drugs which were discontinued were reintroduced, and metronidazole was given again, and within less than 24 hours the patient developed the same psychiatric symptoms. Now the psychiatric symptoms were considered to be caused by metronidazole, and to avoid prolonged administration of metronidazole since hepatic abscess could take a long time to heal if only conservative treatment is undertaken, hepatic abscess drainage was planned so as to decrease the abscess volume which would help in the faster recovery. Consequently, hepatic abscess drainage was performed by the interventional radiologist and now only minimal traces of the abscess were left behind. After abscess drainage, to treat the trace amounts of the leftover pus, metronidazole administration was pursued for another seven days and the psychiatric symptoms are present throughout the period when metronidazole is prescribed. After seven days now metronidazole was discontinued after satisfactory clearance and reduction of the remaining abscess on a repeat USG and the patient was discharged home on haloperidol and lorazepam to control the psychiatric symptoms. After a period of 7-10 days of discontinuing metronidazole, the psychiatric symptoms of the patient start to wean off, and both the drugs haloperidol as well as lorazepam were tapered down and stopped. The patient's physician-daughter got serial USG and lab investigations after the discharge on days 10, 30, 60, and 90 which were normal, and a follow-up for a period of 10 months with no remissions. 

This case is highly unusual because documented recrudescence of symptoms occurred with drug re-administration; thus, this case may satisfy WHO criteria for definite rather than probable or possible causality, rare in single cases of putative drug reactions. Symptoms here are of acute onset, manic-psychotic episodes are temporally related to the use of metronidazole and are self-limiting on discontinuing the drug. Prompt recurrence of symptoms that occur on readministering is striking. Although antibiomania is rare it should be considered with the new onset of acute mania.

## Discussion

We have discussed a case of metronidazole induced mania here. Metronidazole is a commonly used antiprotozoal and antibacterial drug with a vast array of uses. It is contemplated as a cost-effective drug with favorable pharmacokinetic properties and some minor side-effects. Metronidazole is a synthetic version of 5-nitroimidazole which is inactive initially when administered and turns into a metabolically active form on reaching the target site. Drug-induced mania is a well-known clinical entity. A number of drugs like isoniazid, alprazolam, thyroxine, captopril, baclofen, ciprofloxacin, cotrimoxazole, erythromycin, etc. are known to cause manic-psychic episodes [8-10]. Although it is a well-established fact that metronidazole can pass the blood-brain barrier, little is known about its central mode of action. Preliminary findings suggest both direct, i.e., toxic, and indirect CNS effects [13].

Instances of mania caused by metronidazole are super-rare and the mechanism by which it causes is still speculative. So far, there have been no reports addressed for the effective treatment of the deteriorating condition after metronidazole intake.

Here, in this patient metronidazole is administered 1.5g/day for 12 days and the average cumulative dose was 18 grams after which the patient started to develop manic symptoms. Neurotoxicity can be caused by metronidazole use which can present with encephalopathy, cerebellar symptoms (ataxia, dysarthria, hypotonia, etc.), paresthesia, etc., are found on using metronidazole. It is believed that symptoms occur typically after prolonged use but symptoms tend to occur at low cumulative doses too. Symptoms would typically be resolved after cessation of the drug in both the scenarios of metronidazole-induced neurotoxicity (MIN) and metronidazole induced manic-psychic syndrome [14]. 

In the 5th edition of the Diagnostic and Statistical Manual of Mental Disorders (DSM), one of the possible precipitating factors causing secondary mania is the use of antibiotics.

Due to the paucity of published case reports and other literature, questions like the possible mechanism of antibiotic-induced mania, any predisposing factors for this type of mania, if there´s a risk of recurrence of mood episodes following an antibiomania that occurs spontaneously still remains unknown and needs to be worked on.

## Conclusions

Antibiotic treatment can be associated with manic/ hypomanic episodes. The pathophysiological mechanism of the drug-induced mania (DIM) is still elusive, and in an event of drug-induced mania, the offending drug should be discontinued and manic symptoms can be treated lege artis. Our case discussed here shows a strong temporal relationship between the onset and remission of mania in connection to the antibiotic course of metronidazole. As antibiotics are increasingly being used for various conditions, the neurotoxic side-effects are becoming more evident but as there are no set guidelines to treat the neurotoxic side-effects, it poses a great challenge for the physicians on how to tackle the condition since neurotoxicity could present with sundry symptoms a set of guidelines for the treatment of drug-induced neurological symptoms should be devised.
